# Lifestyle-Related Factors in the Self-Management of Chemotherapy-Induced Peripheral Neuropathy in Colorectal Cancer: A Systematic Review

**DOI:** 10.1155/2017/7916031

**Published:** 2017-03-16

**Authors:** Tess M. E. Derksen, Martijn J. L. Bours, Floortje Mols, Matty P. Weijenberg

**Affiliations:** ^1^Department of Epidemiology, School for Oncology and Developmental Biology (GROW), Maastricht University, Maastricht, Netherlands; ^2^Department of Medical and Clinical Psychology, Tilburg University, Tilburg, Netherlands; ^3^Netherlands Comprehensive Cancer Organization, Netherlands Cancer Registry, Eindhoven, Netherlands

## Abstract

*Background.* Chemotherapy-induced peripheral neuropathy (CIPN) is a common adverse effect of chemotherapy treatment in colorectal cancer (CRC), negatively affecting the daily functioning and quality of life of CRC patients. Currently, there are no established treatments to prevent or reduce CIPN. The purpose of this systematic review was to identify lifestyle-related factors that can aid in preventing or reducing CIPN, as such factors may promote self-management options for CRC patients suffering from CIPN.* Methods.* A literature search was conducted through PubMed, Embase, and Google Scholar. Original research articles investigating oxaliplatin-related CIPN in CRC were eligible for inclusion.* Results.* In total, 22 articles were included, which suggested that dietary supplements, such as antioxidants and herbal extracts, as well as physical exercise and complementary therapies, such as acupuncture, may have beneficial effects on preventing or reducing CIPN symptoms. However, many of the reviewed articles presented various limitations, including small sample sizes and heterogeneity in study design and measurements of CIPN.* Conclusions.* No strong conclusions can be drawn regarding the role of lifestyle-related factors in the management of CIPN in CRC patients. Certain dietary supplements and physical exercise may be beneficial for the management of CIPN, but further research is warranted.

## 1. Introduction

Colorectal cancer (CRC) is a highly prevalent type of cancer and a common cancer-related cause of death worldwide [1]. In the past decades, an increase in survival and prevalence rates has resulted from the development of novel treatments and improved diagnostic methods, including early detection strategies through the implementation of population screening programmes. Consequently, more people nowadays survive longer after a CRC diagnosis and live with the physical and mental consequences of the disease and the adverse effects of its treatment [2–4].

CRC is treated through surgery in most cases, combined with (neo)adjuvant chemotherapy and/or radiotherapy depending on the cancer stage [5]. It is known that chemotherapy can cause severe adverse effects that can be highly detrimental to a patient both physiologically and psychologically, thus having a large impact on an individual's quality of life [6]. One common and severe adverse effect of chemotherapy is chemotherapy-induced peripheral neuropathy (CIPN), which usually consists of sensory axonopathies [6]. Although it can depend on the specific affected site of the peripheral nervous system, common signs and symptoms of CIPN usually include numbness and tingling of hands/feet, muscle weakness, lancinating/burning pain, cutaneous hyperesthesia, and loss of pain/temperature sensation [6]. CIPN may develop weeks to months after exposure to chemotherapeutic agents, can continue in spite of cessation of chemotherapy, and may be irreversible depending on the degree of neuron damage [6]. The chemotherapeutic agents which most frequently cause CIPN are thalidomide, bortezomib, platinum derivatives, vinca alkaloids, and taxanes [7].

Platinum-based chemotherapy is the standard treatment for stage III-IV CRC, and it may also be recommended to high-risk stage II patients [5, 8, 9]. Oxaliplatin, a cytotoxic platinum compound which has been used in Europe since 1996, is one of the chemotherapeutic agents used in CRC [10]. Around 60% of CRC patients will receive oxaliplatin-based chemotherapy as part of their cancer treatment. Approximately 48% of patients receiving oxaliplatin-based chemotherapy will develop neuropathy symptoms during treatment, which may resolve after treatment or develop into chronic symptoms in 20–50% of these patients (a phenomenon known as “coasting”) [11, 12]. Another chemotherapeutic agent is cisplatin [13]. Platinum compounds form so-called platinum complexes which inhibit DNA replication, thus leading to apoptosis [7, 14]. It is believed that acute oxaliplatin-related CIPN may be the result of rapid chelation of calcium by oxaliplatin-induced oxalate and that oxaliplatin is able to alter voltage-gated sodium channels, most likely through a pathway involving calcium ions [14]. Another mechanism that has been postulated to contribute to the development of CIPN is a decrease in cellular metabolism in dorsal root ganglion cells [14]. Possible risk factors associated with oxaliplatin-related CIPN include dosage, cumulative dose, treatment schedule, and the patient's muscle mass [14, 15].

An increasing number of CRC survivors suffer from the negative and disabling consequences that CIPN has on their quality of life [3]. Currently, there are no effective treatment options for preventing CIPN or decreasing its symptoms. Neuroprotective agents such as thiols, neurotrophic factors, and anticonvulsants have been tested as possible preventive agents, but no conclusive results have been published [16]. For this reason, research is now also focusing on the potential of lifestyle factors for reducing or preventing CIPN [3]. These lifestyle factors refer to health-related behaviors, such as physical exercise, dietary habits, and intake of dietary supplements, as well as the use of natural products or complementary therapies which patients* choose* to engage in to self-manage their CIPN symptoms, without necessary physician supervision. Previous research has suggested that lifestyle factors, such as an unhealthy diet and body composition, play an important role in CRC development and also affect the health-related quality of life of CRC survivors [1, 17]. For this reason, it is of interest to investigate whether such lifestyle factors could also play a role in the development and consequences of CIPN in CRC survivors.

Although a recent systematic review by Brami et al. [18] reported evidence from randomized clinical trials (RCTs) on effects of natural products and complementary therapies on CIPN in general, the focus of the present systematic review was to evaluate available evidence from research on lifestyle-related factors and their effects specifically on oxaliplatin-related CIPN in CRC. Therefore, the aim of this systematic literature review was to identify lifestyle factors that play a role in the development and occurrence of CIPN symptoms. Such factors could provide avenues for reducing the occurrence of CIPN in CRC patients as well as for promoting self-management options for CRC survivors suffering from chronic CIPN symptoms.

## 2. Methods

For the purpose of this review, a systematic search of the literature was conducted through PubMed, Embase, and Google Scholar. The initial literature search, limited to the time period between 1994 and 2015, was performed on December 8, 2015, by the use of the following search strategy: ((colon OR rectum OR colorectal) AND (cancer OR neoplasm) AND (neuropathy OR peripheral neuropathy) AND (chemotherapy OR platin) AND (lifestyle OR behavior^*∗*^ OR management OR supplement^*∗*^ OR vitamin^*∗*^ OR mineral^*∗*^ OR alternative therapy OR complementary therapy OR BMI OR exercise OR physical activity)).

### 2.1. Article Selection

**Figure 1:**
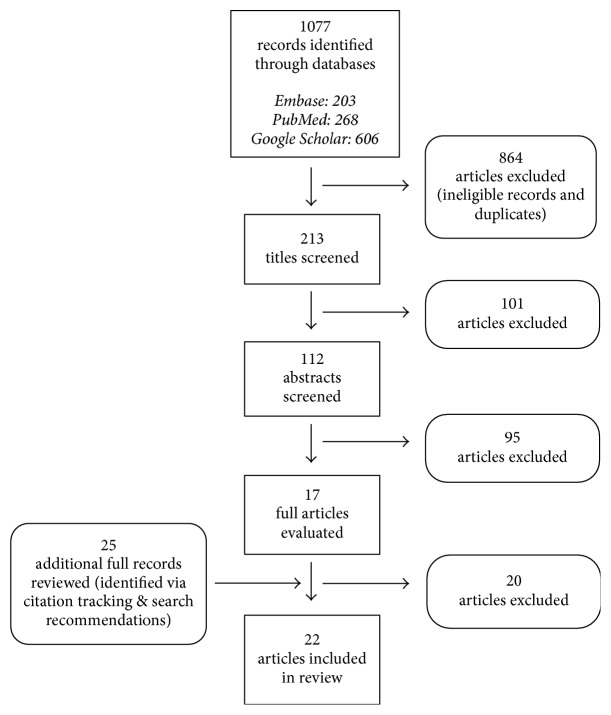
Flow chart showing results of literature search and article screening and selection.

A total of 1,077 records were identified in the initial search (Figure 1). Only original research articles describing the prevention and/or management of oxaliplatin-related CIPN in colon, rectum, or CRC patients were eligible for inclusion. All types of study designs were eligible, except for case reports because of the low level of evidence provided by these studies. For studies containing mixed cancer populations that included CRC, the results of CIPN among CRC patients had to be reported separately from the results of CIPN among patients with other tumors to be eligible for inclusion. Furthermore, articles were required to describe lifestyle-related or alternative/complementary management options for CIPN. For this reason, studies describing prescription medication were not eligible. Studies in which dietary supplements were administered intravenously were also ineligible, and so were animal or cell studies. Finally, studies were only included if the publication was an original research article (e.g., no systematic reviews, book chapters, dissertations, poster abstracts, editorials, and letters to the editor) and if the study had been published in a peer-reviewed journal in English. On the basis of these criteria, 17 full-text articles deriving from the initial search were evaluated for eligibility. An additional 25 articles were identified through citation tracking and screened for eligibility (Figure 1).

## 3. Results

### 3.1. Study Characteristics

A total of 22 articles were included in the review, all of which were published between 1994 and 2015 (see Table 1 for a summary of study characteristics and main findings per article) [3, 11, 19–38]. The majority of the articles reviewed reported results of randomized trials (*N* = 11) [19–21, 23, 24, 26–28, 31–33]. Other research designs included nonrandomized trials (*N* = 5) [22, 34–37], cross-sectional studies (*N* = 2) [3, 11], cohort studies (*N* = 2) [25, 29], case series (*N* = 1) [38], and a crossover trial (*N* = 1) [30]. Furthermore, the study populations were comprised of (exclusively) colon cancer or CRC patients (*N* = 15) [3, 11, 21, 22, 24–29, 31–34, 38] and mixed cancer patients, including CRC (*N* = 7) [19, 20, 23, 30, 35–37].

### 3.2. Measures of Chemotherapy-Induced Peripheral Neuropathy (CIPN)

Overall, the articles presented a wide variety of measures to assess CIPN. Six studies made use of the National Central Cancer Institute Common Terminology Criteria for Adverse Events (NCI-CTCAE v.3 or v.4) [19, 20, 26, 29, 36, 38]. Four studies graded CIPN according to the National Cancer Institute Common Toxicity Criteria (NCI-CTC) [24, 28, 31, 33], two other studies applied the World Health Organization Toxicity Criteria [30, 32], and further three studies implemented the Neurotoxicity Criteria of Debiopharm (DEB-NTC) [25, 27, 28]. One study used a symptom experience diary questionnaire for peripheral neuropathy assessment [21]. Moreover, two studies used the Functional Assessment of Cancer Therapy/Gynecologic Oncology Group-Neurotoxicity (FACT/GOG-Ntx) questionnaire [23, 36], two other studies used the Chemotherapy-Induced Peripheral Neuropathy Assessment Tool (CIPNAT) [11, 34], and another two studies used the European Organisation for Research and Treatment of Cancer Quality of Life Questionnaire, Chemotherapy-Induced Peripheral Neuropathy scale (EORTC QLQ-CIPN20) [3, 35]. Another method to evaluate CIPN included measurements of nerve conduction velocity [37]. Finally, one study did not specify the CIPN assessment measures that had been used [22].

### 3.3. Lifestyle Factors

Results are presented in Table 1. The lifestyle factors identified through the literature search were grouped into four categories: (I) dietary supplements, (II) physical activity, (III) alternative (complementary) therapies, and (IV) multiple/other strategies.

*(I) Dietary Supplements.* The most researched natural remedy for CIPN was Goshajinkigan (GJG or TJ-107), a traditional Japanese herbal extract (“Kampo formula”). Four included studies evaluated the effects of oral administration of GJG and concluded that administering a minimum of 2.5 grams of GJG during chemotherapy significantly reduced the toxic effects of oxaliplatin, reducing the incidence of CIPN and its intensity [25–27, 29]. More specifically, Kono et al. [25] found that the incidence of CIPN symptoms at higher doses of oxaliplatin was lower in the groups receiving GJG. At the same time, patients receiving GJG tolerated higher cumulative doses of oxaliplatin [25]. A study by Nishioka et al. [27] found a significant reduction of grade III CIPN for patients receiving GJG. Finally, Yoshida et al. [29] observed a significant difference in the development of CIPN between GJG and control patients, with less than 10% of GJG patients presenting CIPN symptoms. In contrast, however, a recent randomized placebo-controlled trial by Oki et al. [28] showed that GJG supplementation (7.5 mg/d) during FOLFOX6 chemotherapy in patients with resected stage III colon cancer led to a significantly earlier time to onset of sensory neuropathy (grade 2 or greater) compared to a placebo group.

A further traditional herbal extract that appeared to have a positive effect on CIPN was the Jianwei Hiangqi Guizhi Wuwu Decoction (JHGWD), a traditional Chinese medicine composed of botanical materials [30]. Administration of JHGWD during oxaliplatin chemotherapy significantly reduced the intensity of CIPN symptoms, as well as their duration [30]. Furthermore, the symptoms presented by control patients were more severe in nature and of longer duration [30]. Another traditional Chinese medicinal remedy studied was guilongtongluofang, an aqueous extract of dried herbs. In their study, Liu et al. [31] found that 200 mL of guilongtongluofang taken twice daily significantly lowered the percentages of grade I and II CIPN after 2 and 6 cycles of chemotherapy. The risk of grade I CIPN was significantly lower in the treatment group, and the onset of CIPN symptoms was significantly later in the treatment group than in the control group (9.4 weeks versus 6.5 weeks, resp.) [31]. Furthermore, a randomized controlled study of fucoidan, a sulfated polysaccharide extracted from brown seaweed, showed that the incidence of peripheral neuropathy in patients with advanced CRC was not significantly different between a fucoidan-treated group and an untreated control group [32].

Three studies investigated the effect of vitamin E (400 mg) on CIPN and found no significant differences between oral administration of vitamin E and placebo [19, 20] or an untreated control group [21]. Contradicting results were found in relation to the effects of the antioxidant alpha-lipoid acid (ALA). In a study by Gedlicka et al. [22], 7 patients suffering from grade II CIPN and 1 patient presenting grade III CIPN showed improvement of at least one grade. In contrast, Guo et al. [23] found no significant differences between ALA and placebo. Another antioxidant, *N*-acetylcysteine, was shown to have beneficial effects in patients undergoing oxaliplatin chemotherapy by reducing the incidence of CIPN, as well as by reducing neurophysiological changes during the course of treatment [24].

Finally, supplementation of the amino acid glutamine in patients undergoing oxaliplatin chemotherapy for metastatic CRC resulted in reduction of grade I and II CIPN following two cycles of therapy and reduction of grade III and IV CIPN after four and six cycles [33]. Patients also reported less CIPN-related interference of their daily activities (7.1% interference for patients receiving glutamine versus 40.9% interference for control patients) [33]. 

*(II) Physical Activity.* Evidence supporting a role of physical activity in alleviating CIPN symptoms was scarce; only two studies were identified. According to a cross-sectional study by Mols et al. [3], CRC survivors who reported to meet the recommended Dutch physical activity guideline of 150 minutes of moderate-to-vigorous physical activity per week also reported less CIPN symptoms. Furthermore, a small single-arm trial on the effectiveness of strength and balance training for CRC patients, who had finished their chemotherapy treatment, showed improved balance and lower extremity strength after four weeks of biweekly 60-minute training sessions, as well as improved neuropathic symptoms [34]. 

*(III) Alternative (Complementary) Therapies.* Some evidence was found for the use of complementary therapies to manage neuropathic symptoms, such as acupuncture. A small pilot study by Ogawa et al. [36] reported improved neuropathic symptoms after 4–6 sessions of contact needle therapy (traditional acupuncture). Another small study observed that acupuncture improved nerve conduction velocity in mixed cancer patients, including CRC, with CIPN [37]. Similarly, a case series of 10 patients receiving traditional Chinese acupuncture showed significant amelioration of CIPN symptoms, as well as lowered incidence of CIPN and reduction of symptom mitigation [38]. A further study tested the effects of Scrambler therapy in patients with CIPN, a method of cutaneous nerve stimulation that aims to relieve pain by providing “nonpain” information to block the action of pain stimuli [35]. Statistically significant improvements were observed in pain perception, motor and sensory scales, and interference with life scores [35]. 

*(IV) Multiple/Other Strategies.* A single cross-sectional study was identified which described multiple self-management strategies applied by stage III-IV CRC patients in order to minimize neuropathic symptoms [11]. A variety of strategies were mentioned by participants, including use of dietary supplements (e.g., B vitamins, ALA, and glutamine), over-the-counter medications (e.g., nonsteroidal anti-inflammatory drugs), and nonpharmacological strategies such as avoiding cold/keeping warm, massaging/rubbing affected areas, and various types of physical activity or exercise (e.g., walking, biking, or yoga) [11]. However, since only qualitative data analyses were performed in this study, no significant relief effects of the reported self-management strategies could be shown.

## 4. Discussion

Currently, there are no established treatments for CIPN. This systematic literature review aimed to identify lifestyle-related factors that could potentially empower CRC patients to decrease the risk of developing CIPN and/or reduce CIPN symptoms, thereby reducing the impact of CIPN on their daily functioning and quality of life. Although 22 eligible studies were identified that described a number of lifestyle-related factors, no conclusive evidence was provided regarding any particular factor. Nevertheless, the findings of this review may offer clues for potentially relevant lifestyle factors that could be further explored in future studies.

There is some weak evidence pointing to beneficial effects of herbal extracts or supplements for managing or preventing CIPN in CRC. In general, most evidence points to traditional medicines, such as GJG, as possible management options for CIPN symptoms [25–27, 29]. This evidence is further supported by animal studies suggesting that GJG may prevent cold hyperalgesia and mechanical allodynia without adversely affecting the antitumor effect of oxaliplatin on tumor cells [39]. However, one study also showed negative effects of GJG on neuropathy [28] and the majority of studies reviewed were limited by design, such as having small sample sizes, and results were difficult to compare because of the use of different methods to measure CIPN. Therefore, the results of these studies should be interpreted with caution. Regarding the discrepancy across articles with regard to the method of measuring CIPN, it is important to note that toxicity scales can vary in the way they score symptoms. A change from “grade II to grade I” CIPN defined according to one scale may not be directly comparable to another study in which a change in CIPN is defined based on a different scale. For this reason, varying CIPN measurement methods may therefore explain why contradictory results have been observed in different studies on the same variable. This could, for instance, have been the case for the contradicting results regarding the effect of ALA on CIPN [22, 23]. Furthermore, although questionnaires and self-reports are the preferred measure of CIPN [16], they can be highly susceptible to social desirability bias and be influenced by the ability of participants to accurately recall and estimate the level of past pain symptoms.

Besides the variation in measurement methods, there was a large variation in sample sizes of the reviewed studies. The largest study included 1,644 CRC survivors [3], while 7 out of the 22 articles reviewed reported findings of studies with less than 20 participants [22, 24, 32, 34, 36–38]. As it is hardly possible to generalize the results obtained in studies with such small sample sizes, future studies in larger samples of CRC patients are needed. Furthermore, 7 articles reported results of studies in mixed cancer samples [19, 20, 23, 30, 35–37]. Although these mixed cancer populations were included in this review since they also contained CRC patients, it is possible that the results of these studies reflected effects of the lifestyle factor of interest on CIPN in patients with cancers other than CRC. The reviewed articles also presented a large variety in study designs. Only 11 out of the 22 articles described results of randomized controlled trials, which would be the preferred design for evaluating effects of lifestyle factors on CIPN.

Though the evidence for a role of lifestyle-related factors in preventing and managing CIPN was weak in general, the results of this review may provide relevant leads for future research. Some areas of further research have already been mentioned, and a few others can be pointed out; especially some of the self-management methods described by patients themselves may warrant further investigation [11]. Factors of interest could be, for example, the use of NSAIDs or B vitamins to alleviate chronic CIPN symptoms. Other studies on CIPN, which did not meet the inclusion criteria for the present review, mentioned meditation, yoga, and Reiki [40], as well as gingko biloba and acetyl-L-carnitine supplementation [41, 42], as possibly relevant lifestyle-related factors. Furthermore, although the studies reviewed did not find any relationship between vitamin E and CIPN in CRC patients treated with oxaliplatin, studies testing the efficacy of this micronutrient to reduce CIPN in cisplatin (a platinum compound similar to oxaliplatin) therapy have shown beneficial effects [43–45]. The relationship between vitamin E and CIPN should thus be studied further. In addition, the study by Lin et al. [24] offers a potentially promising treatment option warranting further investigation, as* N*-acetylcysteine naturally increases blood levels of glutathione, which is another antioxidant that has also been shown to possess neuroprotective qualities when administered intravenously during oxaliplatin therapy [46, 47]. Moreover, the evidence that glutamine could help manage CIPN has been further supported by animal studies and intravenous administration of the amino acid [48, 49]. Finally, it might be interesting to investigate the role of calcium and magnesium supplementation, as suggested by the findings of a case report [50] and also because some evidence exists regarding the role of calcium and magnesium infusions on platinum-related CIPN [51–54].

Somewhat unexpectedly, evidence regarding the role of physical activity on CIPN was found to be limited in CRC survivors. There is evidence that physical activity reduces CIPN in breast cancer patients, ovarian cancer patients, and patients with diabetic peripheral neuropathy [55–59]. For this reason, further studies in CRC survivors are highly needed to examine the effects of physical activity on CIPN, especially since physical activity can have beneficial effects on many health-related outcomes in CRC survivors. Moreover, although the evidence concerning CRC is limited, evidence suggests that acupuncture can reduce the symptoms of non-CRC CIPN, diabetic peripheral neuropathy, and neuropathic symptoms experienced by HIV-positive patients [60–63]. It could therefore be relevant to further investigate acupuncture treatments, as such traditional medicine might offer patients a drug-free coping mechanism for neuropathic pain. Lastly, animal studies have provided insight into other factors/supplements which could be worth studying in CRC patients, such as curcumin, flavonoids, reduced poly-amine diets, and vitamin C, all of which have been found to decrease CIPN in mice models [64–68].

## 5. Conclusion

The results of this systematic review indicate that evidence regarding lifestyle-related factors and their potential for the prevention and management of CIPN is very limited. The studies identified in this review might suggest a possible role for supplements such as herbal extracts, physical activity, and alternative therapies such as acupuncture in the self-management of CIPN. Further research with stronger study designs, larger samples, and uniform measures of CIPN is pivotal to confirm such claims.

Taken together, the studies reviewed are inconclusive regarding a possible role for lifestyle-related factors in the management of CIPN in CRC. Nevertheless, this systematic review can provide potential guidance for future studies concerning the role of lifestyle factors in the development and management of CIPN in CRC.

## Figures and Tables

**Table 1:** General summary of articles included in the review.

Reference & country	Sample characteristics	Study design	Lifestyle factor	Exposure	Treatment	Measure of peripheral neuropathy	Summary of results
*Dietary Supplement*							

Afonseca et al., 2013 [19] Brazil	34 patients (18 male, 16 female) diagnosed with gastric cancer (8) or CRC (26) Average age: 56.6 y/o	Phase II, prospective, randomized, placebo-controlled pilot study	Antioxidant: vitamin E	400 mg of vitamin E or placebo orally administered twice daily, starting 5 days prior to treatment (length not specified), plus 1 g (each) of calcium gluconate and magnesium sulfate IV for 30 mins prior and following oxaliplatin infusion	FLOX, FOLFOX, EOX, or XELOX	Assessed by the NCI-CTCAE v3.0	No significant decreases in the incidence of acute oxaliplatin-induced PN in the experimental group compared to the control.

Kottschade et al., 2011 [20] United States	207 patients (155 female, 34 male) scheduled to undergo curative-intent chemotherapy, including CRC patients	Phase III randomized-placebo controlled clinical trial	Antioxidant: vitamin E	400 mg of vitamin E (*d*l-alpha-tocopherol) or placebo twice daily (orally administered) starting 4 days prior to chemotherapy until 1 month after chemotherapy	Patients received either taxane (109 patients), cisplatin (8), carboplatin (2), oxaliplatin (50), or combination (20) chemotherapy	CTCAE v3.0. North Central Cancer Treatment Group (NCCTG), a diary and questionnaires (at baseline, prior to each treatment and at 1 and 6 months succeeding chemotherapy completion)	No significant differences between the experimental and control group.

Salehi and Roayaei, 2015 [21] Iran	65 patients (41 male, 24 female) with CRC Mean age: 56 y (vitamin E group, *n* = 32) and 59 y (control, *n* = 33)	Randomized controlled clinical trial	Antioxidant: vitamin E	400 mg/day of vitamin E, starting within 4 days of the beginning of chemotherapy	FOLFOX4 regimen	Symptom experience diary questionnaire, completed at baseline and after sixth course of chemotherapy	No significant difference in mean peripheral neuropathy score changes between vitamin E and an untreated control group.

Gedlicka et al., 2002 [22] Austria	15 CRC patients (11 male, 4 female) Mean age: 65 y	Pilot trial	Antioxidant: alpha-lipoic acid	600 mg of IV ALA, administered once a week for 3–5 weeks, followed by 600 mg of oral ALA until recovery from neuropathic symptoms, for a maximum of 6 months	130 mg/m^2^ oxaliplatin in combination with 3 mg/m^2^ of raltitrexed every 3 weeks Patients received a median of 6 chemotherapy courses and a mean oxaliplatin (cumulative) dose of 780 mg/m^2^	Unspecified peripheral neuropathy grading scale	Symptoms improved in 8 patients. The average treatment with ALA was 2 months and the mean response time was 4 weeks.

Guo et al., 2014 [23] United States	247 cancer patients (176 CRC), 129 male & 118 female. Mean age: 56 y	Randomized, double-blind, placebo-controlled trial	Antioxidant: alpha-lipoic acid	600 mg oral ALA, 3x daily, or placebo for 24 weeks during chemotherapy (except during the period between 2 days before and 4 days after chemotherapy)	Nonplatinum exposure, cisplatin < 399 mg/m^2^ or oxaliplatin < 750 mg/m^2^; cisplatin > 400 mg/m^2^ or oxaliplatin > 750 mg/m^2^	FACT/GOG-Ntx sub-scale, version 4, at baseline, 24, 36, and 48 weeks of treatment	Oral ALA administration was ineffective at preventing CIPN.

Lin et al., 2006 [24] China	14 stage III colon cancer patients (9 male, 5 female) with a minimum of 4 lymph node metastases Mean age: 60 y	Randomized, placebo-controlled pilot study	Antioxidant: *N*-acetylcysteine	1200 mg of oral *N-*acetylcysteine or placebo administered 1.5 hrs prior to chemotherapy treatment	Biweekly 85 mg/m^2^ oxaliplatin + weekly 5-FU (425 mg/m^2^) bolus and 20 mg/m^2^ LV dose	Assessed every 2 weeks with the National Cancer Institute, common toxicity criteria (NCI-CTC) Patients examined electrophysiologically after 4, 8, and 12 cycles of therapy	Significantly reduced experience of CIPN for patients in experimental group. No electrophysiological changes in the experimental group.

Kono et al., 2011 [25] Japan	90 patients (52 male, 38 female) with metastatic CRC. Average ages: 62 (Group A), 61.5 (Group B), 63 (Group C), 64 (Group D)	Retrospective analysis of intervention study	Herbal extract (traditional medicine): Goshajinkigan (GJG or TJ-107)	7.5 mg/day of GJG administered orally before or in between meals in 2-3 doses during therapy 1 g of IV Ca and Mg (each) prior to and after therapy (Groups B & C)	FOLFOX4 or FOLFOX6 regimens	Evaluations based on the Neurotoxicity Criteria of Debiopharm Assessment of PN in relation to oxaliplatin (total dose) was based on Kaplan-Meier analyses	CIPN grade 1, 2 or worse occurred less regularly in Group A (chemotherapy + GJG), however the results were not significant. CIPN of grade 3 did not occur in any of the groups receiving GJG (Groups A and C). GJG reduced toxicity in patients receiving oxaliplatin chemotherapy.

Kono et al., 2013 [26] Japan	89 patients (48 male, 41 female) with histologically confirmed CRC Mean ages of 67 y (experimental group) and 61 y (control)	Phase II, randomized, double-blind, placebo-controlled study	Herbal extract (traditional medicine): Goshajinkigan (GJG or TJ-107)	GJG powder (2.5 g) or placebo administered orally 3 times/day prior to meal starting on first day of oxaliplatin infusion for 26 weeks	FOLFOX4 or mFOLFOX6 regimens	Assessed at baseline and every two weeks until 8th cycle, succeeded by assessment every 4 weeks until week 26, according to NCI-CTCAE v3.0	Incidence of grade 2 or 3 oxaliplatin-induced PN was significantly lower in experimental groups compared to controls. GJG may serve to delay PN.

Nishioka et al., 2011 [27] Japan	45 patients (22 male, 23 female) with advanced of recurrent CRC Mean age: 67 y (experimental group) and 65 y (control)	Randomized, placebo-controlled study	Herbal extract (traditional medicine): Goshajinkigan (GJG or TJ-107)	7.5 mg/day of GJG or placebo administered orally before or in between meals in 2-3 doses during therapy	FOLFOX6	Patients assessed at baseline and prior to each treatment. CIPN evaluations were based upon the Neurotoxicity Criteria of Debiopharm (DEB-NTC)	Incidence of grade III CIPN was significantly lower in GJG group but there were no significant differences in grade I or II CIPN. The percentage of PN of grade II and III was lower in the experimental group compared to the control.

Oki et al., 2015 [28] Japan	182 patients (99 male, 83 female) with resected stage III colon cancer. Mean age: 62 y (experimental group, *n* = 89) and 60 y (control, *n* = 93)	Randomized, placebo-controlled study	Herbal extract (traditional medicine): Goshajinkigan (GJG)	7.5 mg/day of GJG or placebo administered orally before or in between meals in 2-3 doses during therapy	FOLFOX6	Primary neuropathy assessed by NCI-CTCAE v3.0 (DEB-NTC was used for comparison) and by standardized questions regarding symptoms of neurotoxicity to classify grade (1–4)	Incidence of grade 2 or greater peripheral neuropathy was 50.6% in GJG group and 31.2% in control group. Time to onset of grade ≥ 2 sensory neuropathy was significantly less in GJG group (hazard ratio: 1.9, *P* = 0.007).

Yoshida et al., 2013 [29] Japan	29 CRC patients (17 male, 12 female) Mean age: 60.4 y	Retrospective analysis of intervention study	Herbal extract (traditional medicine): Goshajinkigan (GJG or TJ-107)	GJG powder (2.5 g) administered orally 3 times/day before or between meals	mFOLFOX6 or XELOX regimens	PN measured at the end of chemotherapy according to the CTCAE v.4	Significant difference between groups in regard to the deleterious effects of PN, with patients in the experimental group suffering from less deleterious PN. Furthermore, the incidence of grade III PN was lower in the experimental group compared to the control.

Yuan et al., 2006 [30] China	31 patients (21 male, 10 female), 23 CRC and 8 gastric cancer. Mean age: 58 y	Randomized, controlled, crossover trial	Herbal extract (traditional medicine): Jiawei Hiangqi Guizhi Wuwu Decoction (JHGWD)	One dose of JHGWD twice decocted in water (to 100 ml), then mixed (200 ml), and divided into two portions taken twice daily, starting at the beginning of chemotherapy until the 21st day of treatment (21-day cycles)	2 cycles of chemotherapy: Day 1 130 mg/m^2^ oxaliplatin (diluted in 500 ml of 5% glucose solution) IV solution Days 1–5 200 mg of IV LV and 500 g 5-FU	PN measured according to the World Health Organization Toxicity Criteria	Significantly stronger and longer neurotoxicity symptoms occurred in the control group compared to the experimental group (87.1% compared to 64.5%).

Liu et al., 2013 [31] China	120 CRC patients (83 male, 37 female) Mean age: 52.5 y	Randomized, double-blind, placebo-controlled trial	Herbal extract (traditional medicine): guilongtongluofang	One dose of the extract once decocted in water to 100 mL and then to 200 mL Dose was then divided into two portions taken twice daily Administered 3 days prior to the start of each course of chemotherapy and continued for 10 consecutive days	6 cycles of FOLFOX4	Neuropathy assessed every 2 (completed) cycles using the NCI-CTC	CIPN significantly lower in the trial group after 2 and 6 cycles (significant difference in the onset of CIPN across groups). Furthermore, the cumulative incidence of grade I and sensory neuropathy was significantly lower in the trial group

Ikeguchi et al., 2011 [32] Japan	20 patients (13 male, 7 female) presenting with advanced, unresectable CRC, or recurrent CRC. Mean ages of 71.3 y (experimental) and 69.6 y (control)	Randomized, controlled trial	Herbal extract: fucoidan	150 ml/day of fucoidan solution (4.05 g fucoidan) for 6 months during chemotherapy	FOLFOX6 or FOLFIRI regimens	PN measured according to the World Health Organization Toxicity Criteria	No significant difference in incidence of peripheral neuropathy between fucoidan and untreated control group

Wang et al., 2007 [33] Taiwan	86 patients (56 male and 30 female), with metastatic CRC	Randomized, controlled trial	Amino acid: glutamine	15 g of levo-glutamine administered orally twice daily for 7 consecutive days every 2 weeks beginning at the start of chemotherapy	85 mg/m^2^ oxaliplatin on days 1 and 15, accompanied by 20 mg/m^2^ of FA over 10–20 mins, succeeded by a 500 mg/m^2^ 5-FU bolus on days 1, 8, and 15 every 28 days	Patients assessed electrophysiologically at baseline and again after 2, 4, and 6 cycles of chemotherapy according to the (NCI-CTC)	Experimental group showed a lower percentage of grade I and II CIPN after two cycles, as well as a significantly lower incidence of grade III and IV CIPN after 4 cycles.

*Physical activity*							

Mols et al., 2015 [3] The Netherlands	1643 CRC cancer survivors (935 male, 708 female) Mean age: 66.7 y/o (chemotherapy group), 70.5 y/o (no chemotherapy group) Average years since diagnosis: 5.6 (chemotherapy group), 6.1 (no chemotherapy group)	Cross-sectional study	Exercise	Average time (hrs/weeks) spent walking, biking, housekeeping, gardening, and in sports	Details not available	EORTC QLQ-CIPN20	Chemotherapy resulted in a significantly larger number of patients reporting CIPN symptoms (despite physical activity). Patients meeting the recommended Dutch physical activity guideline of 150 mins/week of moderate to vigorous exercise reported significantly less CIPN.

Tofthagen et al., 2014 [34] United States	4 (2 male, 2 female) CRC patients, having completed oxaliplatin-based chemotherapy 6 months prior to recruitment Age: 80 y/o (participant 1), 66 y/o (participant 2), and 61 y/o (participant 3), age of fourth participant unspecified	Pilot, single-group intervention study	Exercise: strength and balance training	Biweekly, 60-minute sessions for 12 weeks Session composed of 20 mins of strength training, 20 mins of balance training, 10 mins of stretching, and 10 mins of functional balance training	Oxaliplatin-based chemotherapy (exact treatment not specified)	CIPNAT and modified version of the Total Neuropathy Score Assessment occurred at baseline and at 4, 8, and 12 weeks	Peripheral neuropathy symptoms were significantly improved over time, according to the TNS. The mean scores for balance and strength also showed improvement over time.

*Alternative (complementary) therapy*							

Coyne et al., 2013 [35] United States	39 patients (16 male, 23 female) presenting CIPN (included CRC patients) Mean age: 56.5 y	Single-arm trial	Scrambler therapy	45-minute daily treatment for 10 consecutive days Stimulus increased to the max. bearable intensity	Patients received different chemotherapy regimens (including oxaliplatin, cisplatin, and carboplatin)	Pain Numeric Rating Scale and QLC-CIPN-20	Significant improvement of neuropathy found for patients suffering from low, average, and intense pain.

Ogawa et al., 2013 [36] Japan	6 patients (3 male, 3 female) with CRC (5) or breast cancer (1) Mean age: 64.3 y	Single-group intervention study	Contact needle therapy (traditional acupuncture)	4–6 treatments (sessions, length of each not specified) in 3 months	All patients received oxaliplatin regimens expect for patient II, who received docetaxel and paclitaxel	Evaluated according to the CTCAE v.3 and by the FACT/GOG-Ntx	All patients showed improvement of PN symptoms.

Schroeder et al., 2012 [37] Germany	6 patients (3 male, 3 female) presenting with colon cancer, lymphoma, and breast or bronchial cancer + 5 control patients (4 male, 1 female) Mean age: 64 y	Nonrandomized controlled pilot study	Acupuncture	Standard traditional Chinese medicine acupuncture guidelines were followed 10, 20-minute sessions (treatments) over 3 months	Varied per patient/cancer: Colon: oxaliplatin, cisplatin Breast: docetaxel/doxorubicin/ cyclophosphamide Bronchial: cisplatin Lymphoma: rituximab/fludarabin/ cyclophosphamide	Nerve conduction velocity (NCV) measured (with a Neuropack-Sigma) at baseline and once again at a 6-month follow-up	Nerve conduction velocity significantly improved in 5 of the 6 experimental patients, compared to one patient in the control group.

Valentine-Davis and Altshuler, 2015 [38] United States	10 patients (5 male, 5 female) presenting with different stages of colon cancer Mean age: 53 y	Retrospective case series	Acupuncture	Acupoints selected individually prior to each session During each session, stainless steel surgical-grade 1/2 to 1′′ (long) needles Needle retention time varied from 10 to 45 minutes (average of 20 mins) No electrostimulation performed	Different per patient Some participants had already completed their courses of chemotherapy while others were still in treatment Nevertheless, all participants underwent oxaliplatin-based chemotherapy	Measured according to the CTCAE v.4.0	All 10 participants showed improvement in CIPN symptoms (in regard to prevention and mitigation of symptoms). Acupuncture plans focused on increasing blood flow to distal points produced the best improvement.

*Multiple/other*							

Tofthagen et al., 2013 [11] United States	111 stage III-IV CRC Age: >18 y/o	Mixed methods, descriptive, cross-sectional study	Multiple, varied per participant, including supplements (B vitamins, oral calcium and magnesium, omega-3 fatty acids, etc.), NSAIDs (acetaminophen, ibuprofen), acupuncture, massage, light therapy, exercise, and keeping warm/avoiding the cold	Details of exposure to lifestyle factors not available	Oxaliplatin-based chemotherapy (1–8 years prior to study, the average being 3 years)	Questionnaires, CIPNAT	Self-management strategies for alleviating peripheral neuropathy symptoms reported by participants included taking supplements/medications, avoiding cold/keeping warm, and various types of physical activity/exercise (e.g., walking, biking, and yoga).

IV: intravenous; LV: leucovorin; CRC: colorectal cancer; FOLFOX: folinic acid, fluorouracil, oxaliplatin; XELOX: oxaliplatin, capecitabine; Bev: bevacizumab; DOC: docetaxel, fluorouracil, cyclophosphamide, epirubicin; PAC: paclitaxel, cyclophosphamide, doxorubicin; PN: peripheral neuropathy; SOX: oxaliplatin, S-1; FOLFIRI: folinic acid, fluorouracil, irinotecan; NSAIDs: nonsteroidal anti-inflammatory drugs.
